# Alzheimer’s disease and its associated risk of bone fractures: a narrative review

**DOI:** 10.3389/fendo.2023.1190762

**Published:** 2023-08-09

**Authors:** Bing-Na Zhou, Qian Zhang, Mei Li

**Affiliations:** Department of Endocrinology, National Health Commission Key Laboratory of Endocrinology, Peking Union Medical College Hospital, Chinese Academy of Medical Sciences and Peking Union Medical College, Beijing, China

**Keywords:** Alzheimer’s disease, bone fracture, pathological mechanisms, prevention, treatment

## Abstract

**Background:**

Alzheimer’s disease (AD) is a neurodegenerative disorder that is the major cause of dementia in the aged population. Recent researches indicate that patients with AD have a significantly increased fracture risk, but the pathological mechanisms are still unclear.

**Objective:**

We systematically reviewed studies regarding bone fracture risk in AD to uncover links between the pathologies of osteoporosis and AD.

**Methods:**

We searched the literature using the databases of PubMed, Web of Science, Embase and Cochrane Library. Studies were included if they evaluated bone fracture risk in AD patients and if they explored the pathogenesis and prevention of bone fractures in these patients.

**Results:**

AD patients had a significantly higher risk of bone fractures than age-matched controls. Multiple factors contributed to the increased risk of bone fractures in AD patients, including the direct effects of amyloid pathology on bone cells, abnormal brain-bone interconnection, Wnt/β-catenin signalling deficits, reduced activity, high risk of falls and frailty, and chronic immune activity. Exercise, prevention of falls and fortified nutrition were beneficial for reducing the fracture risk in AD patients. However, the efficacy of anti-osteoporotic agents in preventing bone fractures should be further evaluated in AD patients as corresponding clinical studies are very scarce.

**Conclusion:**

Alzheimer’s disease patients have increased bone fracture risk and decreased bone mineral density owing to multiple factors. Assessment of anti-osteoporotic agents’ efficacy in preventing bone fractures of AD patients is urgently needed.

## Highlights

- Bone fractures risk is significantly increased in patients with Alzheimer’s disease (AD)
- The mechanisms include the effects of amyloid pathology on bone cells
- Brain-bone interconnections and Wnt/β-catenin signalling deficits play important roles
- Reduced activity, high risk of falls and frailty increase fracture risk
- Reducing fracture risk is important for patients with AD

## 1 Introduction

Alzheimer’s disease (AD) is a neurodegenerative disease that causes cognitive impairment, of which short-time period memory deficits are a common manifestation (1). Deficits in speech, visuospatial processing and executive function can be presented in AD patients (1). In recent years, the quantity of patients with AD has been growing substantially, accounting for greater than 50% of dementia cases (2). The prevalence of dementia is predicted to increase from 57.4 million individuals worldwide in 2019 to 152.8 million individuals by 2050 (3). The global economic burden of AD and related dementias (ADRDs) is expected to grow rapidly from an estimated $2.8 trillion in 2019 to $16.9 trillion in 2050 (4).

Recently, studies indicate that AD patients have a higher risk of bone fracture, particularly hip fracture, than the older populations without AD (5–7). A meta-analysis including five studies indicated that AD was correlated with a 2.5-fold increased risk of hip fractures (8), which would lead to devastating consequences in AD patients, including loss of function and mobility, reduced quality of life(QOL), prolonged stay for hospitalization, and increased morbidity and mortality (6, 9). The increased falls owing to gait disturbances and postural instability may partially explain the increased risk of bone fractures in AD patients (10, 11). However, other reasons should also be considered for the increased bone fracture risk in AD patients, which deserves further in-depth investigation.

Therefore, we systematically review the studies regarding bone fractures in AD patients and focus on progress in pathological mechanisms and prevention of bone fractures in AD patients.

## 2 Literature search

We systematically reviewed studies about the risk of bone fractures in AD patients, and we conducted a literature search using the databases of PubMed, Web of Science, Embase and Cochrane Library from January 1990 to December 2022, using the keywords ‘fractures’, ‘bone fractures’, ‘broken bones’, ‘osteoporotic fractures’, ‘hip fracture’, ‘Alzheimer’s disease’, ‘Alzheimer-type dementia’, ‘Alzheimer sclerosis’ and ‘Alzheimer syndrome’. In addition, the reference lists of included studies were checked and the names of authors were searched for additional studies. All articles were screened based on their title and abstract. Studies were included if they assessed the risk of bone fractures in AD patients, and studies were also included if they explored the pathogenesis and prevention of bone fractures in AD patients. Articles written in languages other than English, expert opinions, case reports and articles without full texts were excluded.

## 3 Results

### 3.1 The risk of bone fractures in patients with AD

**Figure 1:**
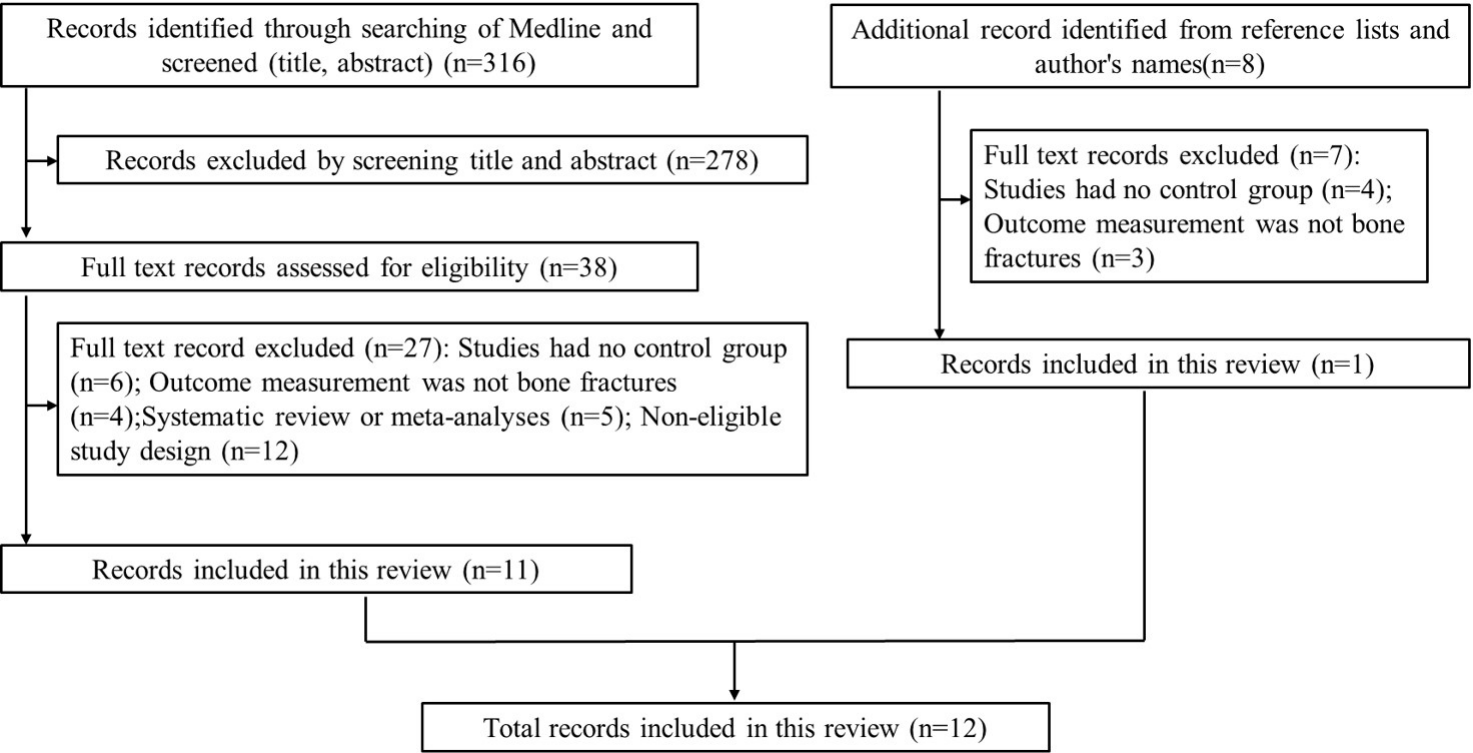
Flow diagram of study selection process.

Our search yielded a total of 316 related studies, and 12 papers met the eligibility criteria (5–7, 12–20) (**Figure 1**). One study was a prospective matched-cohort study, and the others were retrospective matched-cohort studies, with follow-up durations ranging from 1 to 11 years. The characteristics of these studies were summarized in **Table 1**.

**Table 1:** Literature about fracture risk in patients with Alzheimer’s disease.

Design	Country	Fracture	AD		Fracture			Follow-up period	HR/OR	95%CI	Study (reference)
		Number	patients	IR/100 PY	Number	Control	IR/100 PY				
Retrospective cohort	Finland	3,709	70,718	1.85	2,561	70,718	1.49	11.0 years	NA	NA	20
Retrospective cohort	Finland	2,221	50,491	1.63	2,711	100,982	0.69	NA	NA	NA	19
Retrospective cohort	Finland	6,347	46,373	1.56	9,843	92,746	0.64	4.0 years	NA	NA	18
Retrospective cohort	Finland	5,264	67,072	2.23	2,643	67,072	0.98	3.0 years	2.35	2.24-2.46	7
Retrospective cohort	Canada	672	21,015	NA	366	18,301	NA	180 days	1.3	1.1-1.5	17
Prospective cohort	Finland	2,016	27,789	NA	845	25,183	NA	4.0 years	2.57	2.32–2.84	16
Retrospective cohort	Taiwan, China	91	936	NA	190	3,744	NA	3.5 years	2.38	2.02–2.80	15
Retrospective cohort	UK	391	10,052	17.4	226	10,052	6.6	2.2 years	3.2	2.4–4.2	6
Retrospective cohort	USA	955	5396	NA	428	5396	NA	2.1 years	1.856	1.62-2.13	14
Retrospective cohort	Canada	31	528	NA	25	985	NA	10.0 years	2.18	1.26–3.79	13
Retrospective cohort	USA	37	549	NA	300	17,079	NA	1.0 year	1.96	1.34–2.87	12
Retrospective cohort	USA	87	543	NA	49	543	NA	10.0 years	2.0	1.4-2.9	5

AD, Alzheimer’s disease; IR/100 PY, Incident rate per 100 person-years; NA, Not available.

Data from prospective and retrospective matched-cohort studies indicated an independent relationship between AD and an increased incidence of bone fractures. Three studies evaluated the incident fracture rate in AD patients (5, 14, 17). They observed that patients with AD had a higher risk of fracture than those without AD. The most frequent fractures were hip fractures and vertebral fractures in AD patients (14). Seven studies used the incidence of hip fracture as the outcome (6, 7, 13, 15, 16, 18, 19), which indicated that AD patients had an increased risk of hip fractures than patients without AD, regardless of age, gender or AD duration. An independent correlation between AD and hip fractures was found, and AD was an independent risk factor for hip fractures. Another study focused on the reasons for hospitalizations in AD patients and discovered that patients with hip fracture were more likely to be hospitalized (12). A matched-cohort study exploring comorbidities and the risk of mortality in AD patients showed that hip fracture was more prevalent in the AD cohort than in the non-AD cohort and that hip fracture was correlated to the mortality risk of AD patients (20).

### 3.2 The pathogenesis of bone fractures in AD patients

**Figure 2:**
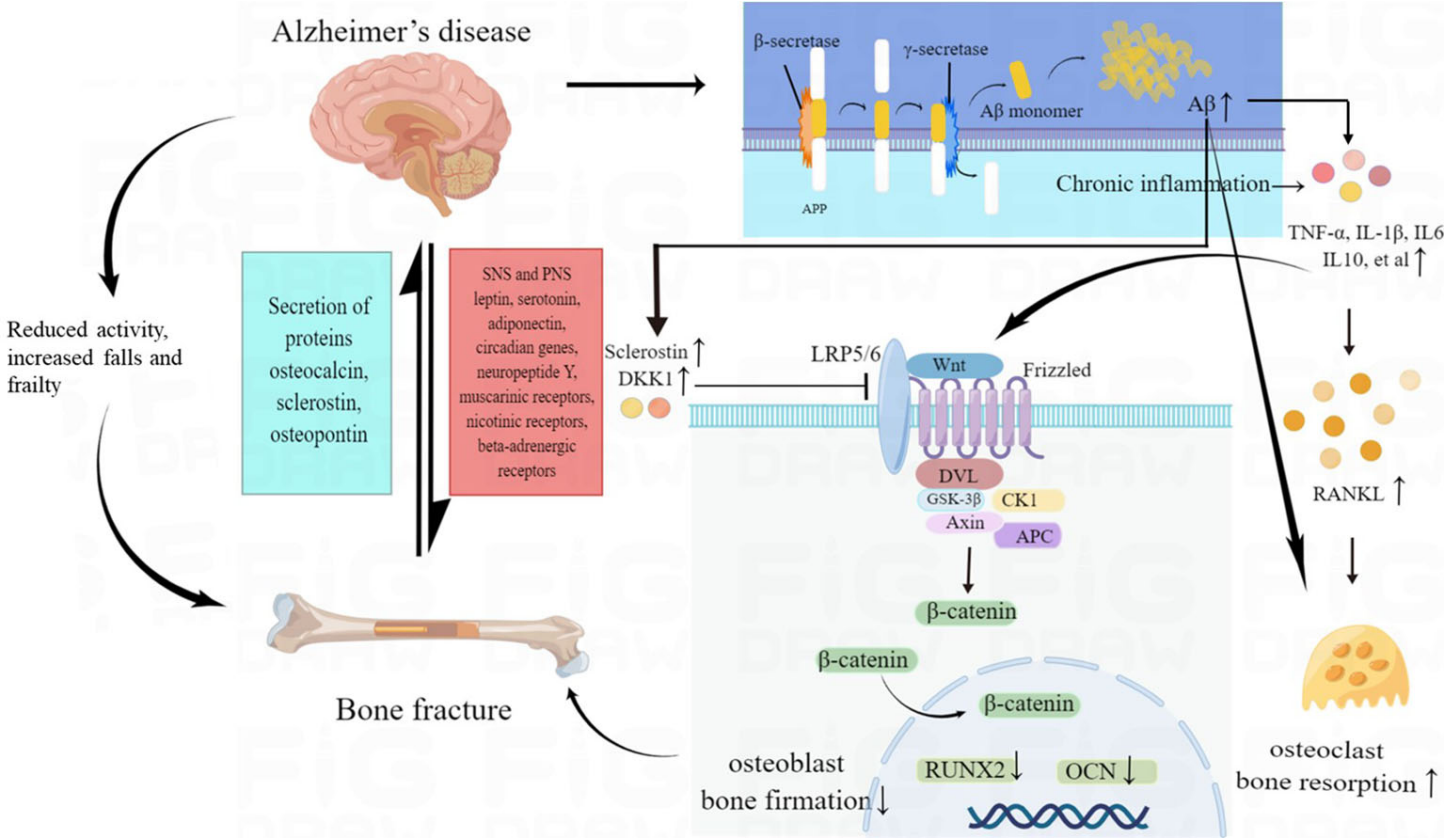
The pathogenesis of increased fracture risk in AD patients. Multiple factors may contribute to bone loss in AD, including direct effects of amyloid pathology on bone cells, abnormal brain-bone interconnection, Wnt/β-catenin signaling deficits, chronic inflammatory status and reduced activity, increased falls and frailty in AD patients. AD, Alzheimer’s disease; Aβ, amyloid-β peptide; SNS, sympathetic nervous systems; PNS, parasympathetic nervous systems; TNF-α, tumor necrosis factor α; IL 1β, interleukin 1β; IL 6, interleukin 6; IL 10, interleukin 10; RANKL, receptor activator of nuclear factorkappa B ligand; RUNX2, runt-related transcription factor 2; OCN, osteocalcin; DKK1, Dickkopf-1.

Studies have found that AD patients often had lower bone mineral density (BMD) than healthy individuals. Low BMD, impaired bone strength and microarchitecture could increase bone fracture risk, which was mainly led by disturbances in bone resorption and bone formation. Multiple factors could contribute to bone loss in AD patients. The pathogenesis of bone fractures in patients with AD was shown in **Figure 2**.

#### 3.2.1 Direct effects of amyloid pathology on bone

Two main pathological hallmarks of AD are the presence of extracellular aggregates of amyloid-beta peptide (Aβ) derived from the transmembrane amyloid precursor protein (APP) and the presence of neurofibrillary protein tangles, which might be composed of hyperphosphorylated tau, withinside the temporal lobe and different cortical areas in the brain which might be related to the death of neuronal cells and synaptic depletion (21). There is developing proof of the involvement of advanced glycation end products (AGEs) in the pathogenesis of AD and their feature as seeds for the aggregation of Aβ (22). The receptor for AGEs (RAGEs) acting as an Aβ binding partner are also included in the pathogenesis of AD (23). Thus, APP/Aβ plays a significant role in the pathogenesis of AD.

Moreover, investigations have suggested that APP/Aβ can directly damage skeletal remodelling by impacting bone cells. A previous study observed that APP and Aβ could regulate *in-vitro* and *in-vivo* osteoclast (OC) differentiation (24). Tg2576 mice, an AD mice model expressing the Swedish mutation of APP (APPswe) below the manipulation of a prion promoter, exhibited biphasic outcomes on OC activation, with an increase in OCs in younger mice but a decrease in older Tg2576 mice (24). In younger Tg2576 mice, the Aβ-RAGE–mediated increase in OCs had a function in selling the discharge of cytokines and elements from the bone matrix, thus included in the pathogenesis of bone fractures. However, in older Tg2576 mice, there was an increase in soluble RAGE (sRAGE) and osteoprotegerin (OPG), causing a decrease in OC formation, which may delay bone remodelling in an unbalanced way and thus might be associated with a higher rate of bone fractures (24). Another study confirmed that a decrease in osteoblastogenesis and loss of trabecular bone mass was resulted from the selective expression of APPswe in mature osteoblast-lineage cells (25). The bone loss was accompanied by elevated adipogenesis and increased bone marrow fat, showing a skeletal aging-like osteoporotic deficit (25). APP could play a physiological role in promoting osteoblast survival and bone formation by preventing oxidative stress and regulating mitochondrial function (26). Mice with knocked out APP gene (APP^-/-^) exhibited osteoporotic-like deficits, including decreased trabecular and cortical bone mass (26). Aβ42 is a key amyloidogenic peptide that is highly associated with AD. Aβ42 can potently enhance osteoclast differentiation and activation but does not affect osteoclast cell viability or number (27). One study observed that 5XFAD mice had decreased volumetric BMD, elevated endocortical bone loss and reduced mineralization with smaller mineral crystals (28). Two pathways were found to be contributing to skeletal fragility in AD *via* alteration of bone quality: accumulation of AGEs and lack of crystallinity, reduced crystal size, and lack of mineralization (28). Another animal study showed that endogenous Aβ might induce osteoporosis *via* mTOR-dependent inhibition of autophagy in bone marrow mesenchymal stem cells (BMSCs) (29). Therefore, APP/Aβ has direct effects on bone cells and may play a vital function in the pathogenesis of bone fractures in AD patients.

#### 3.2.2 The brain-bone interconnection

Recently, the roles of neural control in bone have been found (30). Both the sympathetic nervous system (SNS) and parasympathetic nervous system (PNS) can influence bone *via* numerous pathways, in which circadian genes, neuropeptide Y, serotonin, leptin, adiponectin, muscarinic receptors, nicotinic receptors, beta-adrenergic receptors, and sensory nerve innervation of bone are involved (31, 32). The nervous system can produce specific neurotransmitters and process peripheral hormonal signals, hence affecting bone homeostasis. This increases the probability that the ability of the brain to regulate bone in AD patients could be compromised and consequently reduce bone mass (33). AD patients usually show increased sympathetic tone and decreased parasympathetic flow, and reduced cholinergic innervation in the elderly (34). AD patients treated with AChE inhibitors exhibited a lower risk of hip fracture and stepped forward bone healing, indicating the damaged parasympathetic signalling affects bone homeostasis and might be a target to improve the bone health of AD patients (35).

An understanding of bone’s effects on the brain has also emerged as studies have revealed the possible underlying mechanisms regarding the brain’s influence on bone. Bone act as an endocrine organ that plays important roles by secreting proteins, including osteocalcin (OCN), osteopontin (OPN), and sclerostin (SOST). Blood bone turnover biomarkers, such as C-terminal fragments of collagen, osteoprotegerin (OPG), and OCN were found to be increased in AD patients, showing their association with osteoporosis (36). The bone-derived OCN can impact the production of neurotransmitters, which affect cognitive function (37). Furthermore, a small number of bone marrow-derived stem cells may have the capacity to migrate to the brain, differentiate into microglia-like cells and accelerate Aβ clearance (38). Thus, the brain and bones could be closely interconnected, and bidirectional signalling between brain and bone tissue may have been involved in an increased risk of fracture in AD patients.

#### 3.2.3 Wnt/β-catenin signalling deficits in AD patients

The Wnt/β-catenin signalling pathway is a rich and complicated network that modulates cell proliferation, migration and differentiation, and Wnt proteins orchestrate numerous short-range cell-to-cell communication in mammals (39). In bone, Wnt/β-catenin signalling contributes to osteoblast differentiation and promotes bone formation (40). In brain, the Wnt/β-catenin signalling pathway promotes the formation of synaptic junctions between neurons and increases neuronal survival (41). The impaired Wnt/β-catenin pathway plays a critical role in the development of AD (42). Insufficient Wnt/β-catenin activation impaired bone remodelling and changed the gene expression associated with Wnt/β-catenin signalling in a mouse model (htau mice), which exhibited both low bone mass and AD-like tauopathy (43). Moreover, increased expression of Dickkopf-1 (DKK1) and SOST were found in the bone of htau mice (43). SOST can encode proteins that antagonize the Wnt/β-catenin pathway at preliminary tiers of signalling, which is associated with a reduction in bone formation (44). DKK1 additionally performs numerous roles in the pathogenesis of AD (45). The expression of DKK1 could be overactivated by the pathological protein Aβ, further suppressing the Wnt pathway and triggering a sequence of downstream consequences that enhance the tau hyperphosphorylation and increase extra poisonous Aβ fragments cleavage, thus perpetuating Wnt disorder and promoting the accumulation of toxic protein (45). These provocative data supported that a systemic motive force which includes Wnt signalling deficits would possibly relate to bone loss and brain pathology in patients with AD.

#### 3.2.4 Chronic inflammation may contribute to AD-related bone loss

Neuroinflammation also plays essential roles in the pathogenesis and progression of AD (46). AD pathogenesis can be promoted by chronic neuroinflammation, which is driven by the overactivation of resident microglial cells, the infiltration of macrophages and the involvement of circulating immune cells (47). Numerous inflammatory markers were verified to be significantly altered in a comparison between AD patients and controls (48), and tumour necrosis factor-alpha (TNF-α) ranges were extensively increased in the cerebrospinal fluid (CSF) and the serum of AD patients than healthy controls and associated with disorder development (49), directly potentiating bone reformation in part through synergistic interactions with RANKL and upregulating osteoclast differentiation, thus promoting bone resorption (50). In addition, TNF-α could inhibit bone formation by indirectly suppressing osteoblast manufacturing and proliferation, which is *via* suppressing the expression of insulin-like growth factor-1, osterix (OSX), Wnt, and runt-related transcription factor 2 (RUNX2) signalling (51–53). The levels of interleukin 1β (IL1β), interleukin 6 (IL6), interleukin 10 (IL10) and TNFα were elevated in the cerebral tissue of AD mice, which indicated proinflammatory cytokines promoted the occurrence of AD (54). Inflammatory cytokines might also accelerate bone loss and increase fracture risks in AD patients (55).

#### 3.2.5 Reduced activity, increased falls and frailty in AD patients

AD patients usually have reduced activity in midlife (56). Bones are constantly motivated by weight-bearing movements and muscle contraction, and they are also sensitive to mechanical strain. Osteocytes and their dendritic connections can sense the fluid float driven by stresses placed upon bone. Osteocytes produce signalling molecules that activate bone remodelling in reaction to these stresses (57). Reduced mechanical strain because of immobilization accelerated bone loss in a time-, intense-dependent manner (58). There is an increased risk of falls and injuries in older patients with cognitive impairment compared with healthy controls. There was an up to eight times greater incidence of falls in patients with dementia than in those without cognitive impairment (59). Falls and fall-associated accidents could cause a two- to three-fold risk of hip fracture, slower recovery rates, increased likelihood of multiplied probability of being positioned into residential care and higher mortality than cognitively healthy peers (60). A study confirmed that cognitive impairment became a critical risk factor for falls in older people in Chinese communities (61).

The previous study showed that frailty is highly prevalent in AD patients with the pooled prevalence of 31.9% (62). Frailty is a clinically detectable syndrome associated with the ageing of multiple physiological systems, which could activate vulnerability, Additionally, frailty is associated with malnutrition, cognitive deterioration, atherosclerosis and sarcopenia, and their diverse metabolic alterations (63). A meta-analysis indicated that frailty was highly correlated with falls, bone fractures, hospitalization, disability, dementia, and death (64). Both frailty and decreased mobility lead to reduced muscle strength, and sarcopenia is common in elderly individuals with cognitive deterioration, which could be a vital risk factor for bone fractures in AD patients (65).

### 3.3 Reduce the risk of bone fractures in AD patients

#### 3.3.1 Physical activity, falls prevention and fortified nutrition

Exercise is beneficial to improve gait, balance, strength and mobility, and executive functions and further reduce falls in the older population (66), and it may postpone the progression of AD in a sustainable and cost-effective manner (67). Physical activity or exercise is also important for keeping bone health, since increasing muscle mass and mechanical stress can prevent bone loss (68, 69). Compared to wild-type mice, the AD mice (3xTg mice) had an increased risk for limb fracture. Treatment with resveratrol, exercise, or both in combination can improve fracture resistance and bone strength (70). A recent study showed that a multimodal exercise program reduced the incidence of falls and improved balance, gait, and BMD in institutionalized patients with AD (71). Falls are common in AD patients, and approximately two-thirds of AD patients fall annually, which is double the risk of falls in cognitively unimpaired elderly individuals (72).

Preventing falls is essential for AD patients, and these patients require multidisciplinary management. According to the World Guidelines for Fall Prevention and Management for Older Adults (73), there are wider benefits to reduce the risk factors for falls (e.g., gait and balance problems), including elevated intrinsic capacities (physical and mental health), functioning and the quality of life. Evaluation of the risk of future falls should be performed by experienced clinicians using existing resources. Multidomain interventions, which were a mixture of interventions tailored to the individual, including implementing strength and balance exercises, reviewing medications, optimizing vision and hearing, addressing foot problems and appropriate footwear, using interventions to cope with concerns about falling, making individual education and environmental modifications and so on, whilst delivered, were valid for downscaling the fall rates of high-risk community-dwelling elderly individuals (73).

AD patients are often malnourished because of physiological and psychological factors (74). Nutrition (e.g., *via* vegetables, fruit, and fish) is critical for optimizing cognition and decreasing fracture risk in AD patients (75). The importance of a balanced diet including protein, vegetables, fruit and minerals has been emphasized for bone health and the prevention of fractures (76). Healthy dietary patterns, such as the Mediterranean diet, could be helpful for AD patients and reduce fracture risk. It is beneficial for the prevention of osteoporosis through the supplementation of key micronutrients for bone, such as calcium and vitamin D (77). Additionally, some clinical studies showed that a higher vitamin D status was related to a decreased risk of AD and all-cause dementia, which has supported the significance of vitamin D in cognitive health (78, 79). Some randomized controlled trials found that biomeasures and cognitive function were improved in AD patients after supplementation of vitamin D (80). Thus, it is essential to maintain sufficient vitamin D concentrations to reduce bone loss and neurocognitive decline.

#### 3.3.2 Anti-osteoporotic treatment

In recent years, great progress has been made in drug treatment for osteoporosis, and the effective agents include antiresorptive, anabolic and dual-action agents. However, there are few studies evaluating the effect of anti-osteoporotic agents on the risk of fracture in AD patients. The worry of side effects would possibly result in hesitation to prescribe these agents to the frailest elderly patients. For instance, patients with dementia were probably more sensitive to the serious side effects of bisphosphonates (BPs) (81).

BPs have been proven to be a key intervention for osteoporosis, and they are considered to be one of the first-line medicinal drugs for preventing osteoporotic fractures (82). Some clinical and preclinical studies have revealed that nitrogen-containing BPs (NBPs) may be potential to alleviate the symptoms of neurological disorders such as brain calcification, AD and Huntington’s disease by targeting the mevalonate pathway (83). In the MEDALZ-2005 cohort, the incidence of BP use was 11.2%, and the median period of BP use was 777 days among AD patients (84). But they didn’t evaluate the effects of BPs in that cohort (84). In a cohort comprised of nursing home residents aged 65 years or older, of which 51% patients had moderate-to-severe cognitive impairment, BPs treatment was correlated with a significant reduction in hip fracture among frail, elderly patients (85).

As a fully human monoclonal antibody targeting the bone resorption mediator RANKL, denosumab is effective in postmenopausal osteoporosis, male osteoporosis and glucocorticoid-induced osteoporosis (86–88). Teriparatide, a recombinant fragment of the human parathyroid hormone, is an anabolic drug that can substantially increase BMD and decrease the incidence of vertebral fractures (89, 90). The effects of denosumab or teriparatide on osteoporosis are still unclear in AD patients. The effects of zoledronic acid, denosumab, and teriparatide for preventing hip fractures were evaluated in frail older patients, and some patients with cognitive impairment were included in that study (91). The study showed that denosumab and zoledronic acid might be as effective as teriparatide for the prevention of hip fractures in frail older patients, but further investigation is required to evaluate their efficacy and safety in AD patients (91).

Romosozumab, a monoclonal antibody to sclerostin, is a new osteoanabolic drug that increases bone formation and decreases bone resorption, which is recommended as preliminary treatment in patients with a very high fracture risk without a history of stroke or myocardial infarction (92). Since circulating DKK1 level was significantly correlated with the annual rate of change in cognition (93), DKK1 could be a novel biomarker and promising therapeutic target for osteoporosis in AD patients. No reports have been made targeting sclerostin or DKK1 in osteoporosis in AD patients.

Above all, few studies have evaluated the effects and safety of anti-osteoporotic drugs on osteoporosis in AD patients. There is an urgent need to conduct research on treatment for osteoporosis in AD patients.

## 4 Conclusion

AD patients usually have a high fracture risk, which will further increase the morbidity and mortality of AD patients. Mechanisms of increased fracture risk are multifactorial in AD patients, including direct effects of amyloid pathology on bone cells, abnormal brain-bone interconnection, Wnt/β-catenin signalling deficits, reduced activity, increased falls and frailty, and chronic inflammatory status. Exercise, prevention of falls, and full nutrition are recommended in AD patients. Further studies are necessary to clarify the efficacy and safety of anti-osteoporotic agents in reducing fracture risk in AD patients.

## Author contributions

ML contributed to conceptualization and study design of the research. B-NZ collected the data, analyzed data, and drafted the manuscript. QZ contributed to supervision and revision. All authors contributed to the article and approved the submitted version.
